# Beryllium Increases the CD14^dim^CD16+ Subset in the Lung of Chronic Beryllium Disease

**DOI:** 10.1371/journal.pone.0117276

**Published:** 2015-02-17

**Authors:** Li Li, Nabeel Hamzeh, May Gillespie, Jill Elliott, Jieru Wang, Eva Brigitte Gottschall, Peggy M. Mroz, Lisa A. Maier

**Affiliations:** 1 Department of Medicine, National Jewish Health, Denver, Colorado, United States of America; 2 Division of Pulmonary and Critical Care Sciences, Department of Medicine, School of Medicine, Denver, Colorado, United States of America; 3 Environmental Occupational Health Department, Colorado School of Public Health, University of Colorado, Denver, Colorado, United States of America; 4 Department of Pediatrics, Division of Pulmonary Medicine, Allergy and Immunology, Children’s Hospital of Pittsburgh of University of Pittsburgh School of Medicine, Pittsburgh, Pennsylvania, United States of America; Wexner Medical Center at The Ohio State University, UNITED STATES

## Abstract

CD14^dim^CD16+ and CD14^bright^CD16+ cells, which compose a minor population of monocytes in human peripheral blood mononuclear cells (PBMC), have been implicated in several inflammatory diseases. The aim of this study was to investigate whether this phenotype was present as a subset of lung infiltrative alveolar macrophages (AMs) in the granulomatous lung disease, chronic beryllium disease (CBD). The monocytes subsets was determined from PBMC cells and bronchoalveolar lavage (BAL) cells from CBD, beryllium sensitized Non-smoker (BeS-NS) and healthy subjects (HS) using flow cytometry. The impact of smoking on the AMs cell phenotype was determined by using BAL cells from BeS smokers (BeS-S). In comparison with the other monocyte subpopulations, CD14^dim^CD16+ cells were at decreased frequency in PBMCs of both BeS-NS and CBD and showed higher HLA-DR expression, compared to HS. The AMs from CBD and BeS-NS demonstrated a CD14^dim^CD16+phenotype, while CD14^bright^CD16+ cells were found at increased frequency in AMs of BeS, compared to HS. Fresh AMs from BeS-NS and CBD demonstrated significantly greater CD16, CD40, CD86 and HLA-DR than HS and BeS-S. The expression of CD16 on AMs from both CBD and BeS-NS was downregulated significantly after 10μM BeSO_4_ stimulation. The phagocytic activity of AMs decreased after 10μM BeSO_4_ treatment in both BeS-NS and CBD, although was altered or reduced in HS and BeS-S. These results suggest that Be increases the CD14^dim^CD16+ subsets in the lung of CBD subjects. We speculate that Be-stimulates the compartmentalization of a more mature CD16+ macrophage phenotype and that in turn these macrophages are a source of Th1 cytokines and chemokines that perpetuate the Be immune response in CBD. The protective effect of cigarette smoking in BeS-S may be due to the low expression of co-stimulatory markers on AMs from smokers as well as the decreased phagocytic function.

## Introduction

Chronic beryllium disease (CBD) develops in up to 16% of individuals exposed to beryllium (Be) and is characterized by granulomatous inflammation and the accumulation of CD4+ T cells in the lung [1]. Using the Be lymphocyte proliferation test (BeLPT) we have identified workers with beryllium sensitization (BeS), demonstrating an immune response to Be with an abnormal BeLPT, but no evidence of CBD [2, 3]. Studies show that Be persists within the lungs of individuals many years after exposure has ceased [4], suggesting a failure to clear Be antigen from the lungs. This retention of Be may perpetuate an ongoing Be-specific immune response in CBD and/ or progression from BeS to CBD. It has been hypothesized that alveolar macrophages may undergo apoptosis upon exposure to Be and that this may contribute to retention of Be in the lungs of those with CBD [4, 5].

In human peripheral blood, monocyte subpopulations with distinct functional properties have been defined by their expression of CD14 and CD16. CD14 is the receptor for complexes of lipopolysaccharide (LPS) and LPS-binding protein [6]. CD16 is the low-affinity receptor for the Fc region of IgG (Fcγ receptor type III [FcγRIII]) and plays an important role in the clearance of immune complexes [7]. Monocyte subsets were initially defined as CD14+CD16+ and CD14++CD16+ based on work in healthy control subjects. CD14+CD16+ monocytes, which compose a minor population of monocytes in human peripheral blood mononuclear cells (PBMC), are more mature than CD14++CD16+ classic monocytes [8, 9]. Previous studies have shown that the number of CD14+CD16+ monocytes are expanded during severe infectious and inflammatory conditions, such as Rheumatoid arthritis (RA) [10], tuberculosis [11], asthma [12] and sarcoidosis [13]. In addition, these monocytes have been implicated in the pathogenesis of several inflammatory diseases, such as RA as they produce higher levels of tumor necrosis factor-α (TNF- α) and IL-1β and preferentially differentiate into macrophages [14]. However, this finding has not been confirmed in other diseases, such as systemic lupus erythematosus (SLE) [15]. Moreover, several investigators demonstrated that CD14+CD16+ cells express surface markers and exhibit functional activity characteristic of dendritic cells [16, 17] and have suggested that CD14+CD16+ cells may differentiate into proinflammatory mononuclear cells. More recent evidence suggests that this subpopulation can be further subdivided into CD14^dim^CD16+ and CD14^bright^CD16+ cells. The CD14^bright^CD16+ monocyte subpopulation has been reported to contain the majority of interleukin-10 (IL-10) producing cells and to produce high levels of proinflammatory cytokines such as TNF- α and IL-1 β [18, 19]. In contrast, CD14^dim^CD16+ monocytes appear to have high migratory capacity but only limited phagocytic potential [20]. So far, this has not been investigated in patients with CBD.

Current studies indicate that alveolar macrophages (AMs) arise from circulating blood monocytes, which colonize the tissues under inflammatory and non-inflammatory states. The AMs may improve function in maintaining homeostasis in the lung. On one hand, they fight against pathogens by activating multiple immunological pathways and serve as the first line of defense, while on the other hand, they manifest an anti-inflammatory response to protect excessive tissue damage. Cigarette smoke (CS) induces an accumulation of macrophages in the terminal airways of the lung and this is noted in alterations of BAL cell numbers and % as well as on histological lung samples from smokers [21]. Numerous epidemiologic studies and biological studies have shown that smoking maybe protective or reduce the development of granulomatous lung disease [22–25]. However, the molecular mechanism by which this occurs is not clear.

Since AMs constitutes an important link between pulmonary innate and adaptive immunity due to their antigen-presenting capacity and ability to express different immunomodulating mediators [26], the migration of monocytes from blood to lung and their differentiation into macrophages may be an important step in disease pathogenesis. However, the role of AMs in CBD pathogenesis, and in the protective effects of smoking on CBD has yet to be fully determined. As altered functional capacity of AMs may be reflected in cell surface antigen expression, we hypothesized that CBD will have a different AMs phenotype compared to BeS Non-smoker (BeS-NS) patients and healthy subjects (HS). In addition, since FcγR cross-linking on macrophages potentially initiates phagocytosis, antigen presentation, antibody-dependent cell-mediated cytotoxicity (ADCC) and release of pro-inflammatory cytokines and mediators of tissue destruction [27], we further hypothesized that some of these functions would differ between CBD, BeS-NS, BeS smokers (BeS-S) and HS, as well as in the lung and blood of these subjects and explain the protective effect of cigarette smoke on CBD. The aim of this study was to investigate the relevance of CD14+CD16+ monocyte phenotype as well as the AMs’ cell surface receptor and function in patients with CBD, BeS-NS, BeS–S with normal lung function and HS.

## Materials and Methods

### Study population

All participants gave informed written consent in accordance with the Declaration of Helsinki, and the study was approved by National Jewish Health (NJH) Institutional Review Board (IRB) for Human Subjects. Twenty patients with a diagnosis of CBD, twenty with BeS-NS and ten BeS-S were enrolled in this study from patients seen clinically at NJH. The diagnosis of CBD was established using previously defined criteria, including the presence of granulomatous inflammation on lung biopsy, and a positive proliferative response of blood and/or BAL cells to BeSO_4_ in vitro. The diagnosis of BeS was established based on a positive proliferative response of blood cells to BeSO_4_ in vitro on the BeLPT, and the absence of granulomatous inflammation or other abnormalities on bronchoscopic lung biopsy. Individuals were considered to be current smokers if they had smoked daily for at least the last 3 months. PBMCs were isolated from 10 HS, 10 CBD, 10 BeS-NS subjects, while BAL cells were isolated from 14 CBD, 14 BeS-NS and 10 BeS-S at NJH. Paired PBMCs and BALs samples where obtained from four of the same CBD subjects, and four BeS-NS subjects and none of the HS subjects. Instead, the BAL cells from six HS were obtained as described previously [28]. Briefly, de-identified donor lungs that were not suitable for transplant were obtained through the National Disease Research Interchange (NDRI, Philadelphia, PA, USA) and the International Institute for the Advancement of Medicine (Edison, NJ, USA). Demographic data was obtained on all subjects as part of our research protocol and from the NDRI for the HS. No CBD smokers were available to participate in this research study, as we have very few subjects with CBD who currently smoke.

### Peripheral blood mononuclear cells (PBMCs)

Peripheral blood mononuclear cells (PBMCs) were isolated by Ficoll density gradient centrifugation. The viability of PBMCs was always >95%, as determined by trypan blue staining. The viable cells were quantified in a Neubauer chamber (Zeiss, Oberkochen, Germany) and leftover cells were cryopreserved in liquid nitrogen.

### Bronchoscopy

Bronchoscopy was performed to obtain bronchoalveolar lavage (BAL) cells from patients, as previously described [29]. Briefly, four 60 ml aliquots of sterile normal saline (at room temperature) were instilled into the airway with a syringe then aspirated using low suction. For each collection from an individual patient the aspirated BAL specimens were pooled, kept on ice, and processed within one hour of collection.

The BAL cells from HS were isolated as described previously [28]. The middle lobe was perfused and lavaged with HEPES-buffered saline containing 2 mM EDTA and then with HEPES-buffered saline alone. The lavage was retained for isolation of AMs and the lavage fluid was centrifuged at 4°C for 10 min and washed in Dulbecco’s modified Eagle’s medium (DMEM).

To obtain AMs, the BAL cells were resuspended in DMEM supplemented with 10 % fetal calf serum (FCS), 2 mM glutamine and antibiotics (100 g/ml streptomycin, 100 U/ml penicillin G) and plated on tissue-culture plates. After a 2 h incubation to allow adherence, the cells were washed with DMEM to remove the non-adherent cells. The purity of isolated AMs was close to 100% after adherence in culture, as measured by CD68 staining.

### Flow cytometry analysis

The AMs and PBMCs were plated at a density of 1×10^6^ /ml for culture with and without BeSO_4_ at 37°C for 24 hours. In fresh cells and those after 24h treatment with 10μM BeSO_4_, the expression of CD14, CD16, CD86, and HLA-DR on monocytes and CD11c, CD14, CD16, CD40, CD80, CD86, HLA-DR on AMs were measured using flow cytometry. Cells were washed in FACS medium (PBS containing 1% BSA) and stained at 4°C for 20 min by antibodies directly conjugated with FITC, PE, PerCP or APC. Thereafter, cells were washed three times with PBS and analysed by FACScalibum (Becton Dickinson, Heidelberg, Germany) using the CellQuest software (Becton Dickinson).

### Phagocytosis Assays

Phagocytosis assays were performed as described previously [30]. In brief, one-millilitre aliquots of BAL cells at a concentration of 1× 10^6^ /ml were adhered to 12-well plates. After 24h treatment with 10μM or 100μM BeSO_4_, the fluid was removed and adherent AMs were placed in 1 ml fresh DMEM medium with 10%FCS. Flash Red carboxylate-modified beads (5mm) were added to the AMs monolayers (at a ratio of 5:1 beads/plated AMs for a total of 5× 10^6^ beads) in 12-well plates for 1 hour at 37°C/5% CO_2_. Phagocytosis of beads was determined by flow cytometry.

### Statistical Analysis

A Welch ANOVA was used to determine the effect of treatment. After the data were checked for significant treatment differences, individual contrasts were calculated to compare treatment means of interest. Normalizing transformations were made in cases where the data was non-Gaussian. When data transformations were unsuccessful, we used suitable nonparametric tests. All other comparisons were made using Wilcoxon rank sum test and the paired student’s t-Test. A p < 0.05 was used to determine statistical significance and all tests were two sided.

## Results

### Demographics of participants

The demographics of the HS, CBD, BeS-NS and BeS-S patients are shown in Table 1. No difference was seen in the age of the HS, CBD, BeS-NS and BeS-S enrolled in this study and the majority were male. CBD subjects had a statistically significant increase in the percentage of BAL lymphocytes compared with BeS-NS patients (median 16, range 4–38.5, versus median 6.7, range 1.5–16.5; P < 0.05) and BeS-S (median 3.95, range 1–25.3, versus median 6.7, range 1.5–16.5; P < 0.05). The total BAL cell lymphocytes counts were not available for the HS. BeS-S subjects had a statistically significant increase in the total BAL WBC count (median 100, range 21–365) compared with BeS-NS patients (median 58.3, range 27.5–66.7).

**Table 1:** Clinical characteristics of BeS and CBD subjects.

	HS blood (n = 10)	BeS-NS blood (n = 10)	CBD blood (n = 10)	NDRI HS BAL (n = 6)	CBD BAL (n = 14)	BeS-NS BAL (n = 14)	BeS-S BAL (n = 10)
Age (yr)	67(55–73)	64(50–73)	64(52–75)	52 (43–57)	60 (49–73)	63 (38–72)	50(40–65)
Gender (M/F)	8/2	9/1	9/1	4/2	12/2	10/4	8/2
Race (W/AA/H)	10/0/0	9/1/0	9/1/0	6/0/0	11/0/3	13/1/0	9/0/1
Smoking status (CS/FS/NS)	0/0/10	0/0/10	0/0/10	0/0/6	0/0/14	0/0/14	10/0/0
BAL cells Lymphocytes (%)	NA	NA	NA	NA	16 (4–38.5)*	6.7 (1.5–16.5)	3.95 (1–25.3)
BAL cells Total WBC count (millions)	NA	NA	NA	NA	44(33–50.1)	58.3 (27.5–66.7)	100(21–365)*

Data are expressed as median (range). W, white; AA, African American; H, Hispanic; CS, current smoker; FS, former smoker, NS, never smoker. *p<0.05

### CD14^dim^CD16+ cells were decreased in fresh PBMCs of BeS and CBD subjects

**Fig 1:**
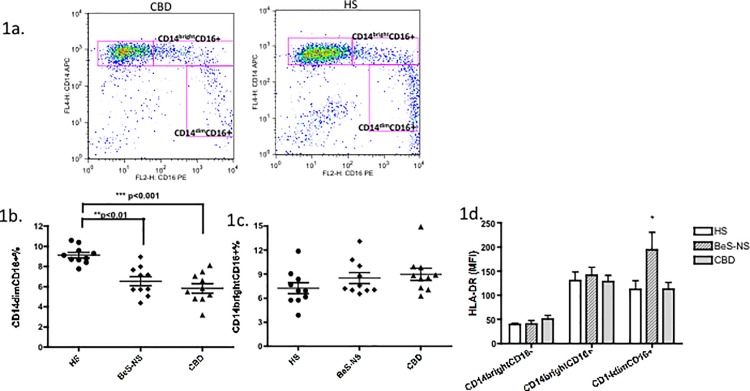
CD14^dim^CD16+ cells were at decreased frequency in fresh PBMCs of both BeS-NS and CBD. 1a. Representative dot plots of CD14 and CD16 expression on monocytes in CBD (Left) and HS (Right). 1b. Percentages of CD14^bright^CD16+monocytes among total monocytes from HS and patients with BeS-NS and CBD. Each dot represents an individual subjects. 1c. Percentages of CD14^dim^CD16+ monocytes among total monocytes from HS and patients with BeS-NS and CBD. 1d. MFI of HLA–DR expression on CD14^bright^CD16-, CD14^bright^CD16+ and CD14^dim^CD16+ monocytes from patients with HS, BeS-NS and CBD. The median percentage is shown as a solid line. MFI = mean fluorescence intensity. *p<0.05.

Frequencies of CD14^bright^CD16+ and CD14^dim^CD16+ cells from PBMCs from 10 CBD, 10 BeS-NS and 10 age and gender matched HS were determined by flow cytometry. The representative staining patterns of surface expression of CD14 and CD16 from a CBD patient and an HS is shown in Fig. 1A. The mean± SD frequency of CD14^dim^CD16+ cells was significantly decreased in CBD patients (5.8±1.5%, p<0.001) and BeS-NS patients (6.5±1.4%, p<0.01) compared to the HS (9.1±0.8%) (Fig. 1B). There was no significant difference in the frequency of CD14^bright^CD16+ cells in CBD (9.0±2.4%) and BeS-NS patients (8.6±2.2%) compared with HS (7.2± 2.2%) (Fig. 1C). In comparison with the classic monocyte CD14^bright^CD16- subpopulations, CD14^bright^CD16+ and CD14^dim^CD16+ cells showed higher HLA-DR mean fluorescent intensity (MFI, Fig. 1D), with the most prominent expression noted in the BeS-NS participants (p<0.05).

### CD14^dim^CD16+ cells were at increased frequency in AMs of CBD, while CD14^bright^CD16+ cells were at increased frequency in AMs of BeS, compared to HS

**Fig 2:**
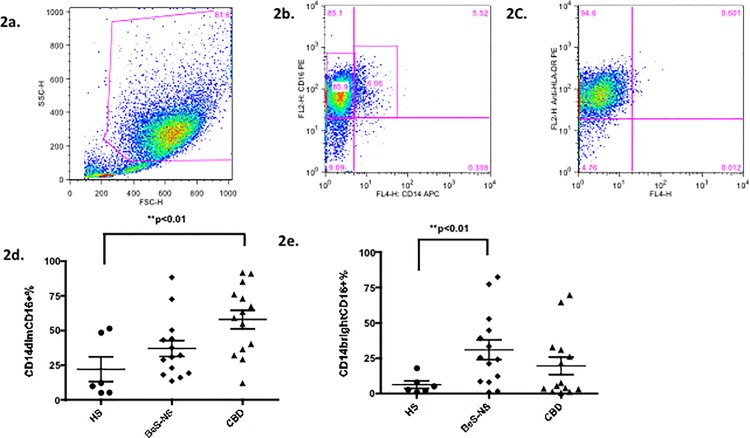
CD14^dim^CD16+ cells were at increased frequency in AMs of CBD, while CD14^bright^CD16+ cells at increased frequency in AMs of BeS. 2a. A representative example of AMs gates from fresh BAL cells from CBD patients. 2b. The expression of CD14 and CD16 using the AMs gate. In the representative examples shown, 85.9% of AM were with a CD14^dim^CD16+ phenotype while only 6.06% of AM with a CD14^bright^CD16+ phenotype. 2c. The expression of HLA-DR using the AMs gate. 95.2% of AMs highly expressed HLA-DR. 2d. Frequency of CD14^dim^CD16+ cells among total AMs from HS and patients with BeS-NS and CBD. Each dot represents an individual subjects. 2e. Frequency of CD14^bright^CD16+ cells among total AMs from HS and patients with BeS-NS and CBD. Each dot represents an individual subjects. **p<0.01.

A representative example of the AMs from CBD demonstrates a CD14^dim^CD16+ phenotype. Specifically, 86% of CBD AMs had a CD14^dim^CD16+ phenotype while only 6% demonstrated a CD14^bright^CD16+ phenotype (Fig. 2A-C). The mean± SD frequency of CD14^dim^CD16+ cells was significantly increased in CBD patients (57.9 ±6.6%, p<0.01) compared to the HS (22.1±8.9%) (Fig. 2D). The mean± SD frequency of CD14^bright^CD16+ cells was significantly increased in BeS patients (30.9±7.0%, p<0.01) compared to the HS (6.2±2.5%) (Fig. 2E).

### Increased expression of CD16, CD40, CD86 and HLA-DR is present on fresh BeS-NS and CBD AMs compared to HS and BeS smokers

**Fig 3:**
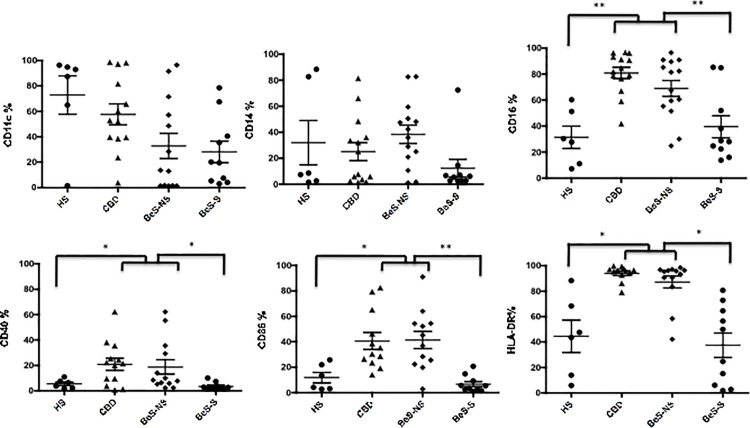
Percent of fresh AMs cells expression CD11c, CD14, CD16, CD40, CD86 and HLA-DR in HS, CBD, BeS-NS and BeS-S. Each dot represents an individual subjects. *p<0.05, **p<0.01.

CBD and BeS-NS AMs demonstrated different cell surface markers in comparison with HS and BeS-S (Fig. 3). Specifically, the expression of CD16 (p<0.01), CD40 (p<0.05), CD86 (p<0.05) and HLA-DR (p<0.05) were significantly increased in CBD and BeS-NS in comparison with HS and BeS-S. No change was detected in the expression of CD11c and CD14 between participants. The AMs expression of CD80 was weak and did not differ between groups (data not shown). There was no significant difference in any of the markers between HS and BeS-S.

### Be exposure increases CD86 on circulating monocytes and decreases CD16 on AMs

**Fig 4:**
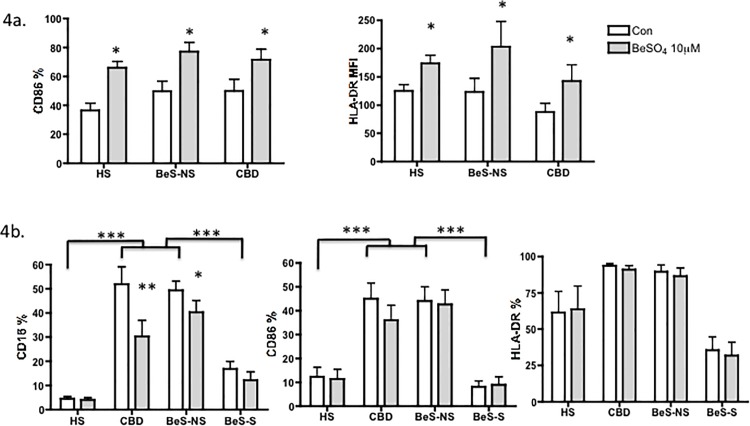
Expression of phenotypic makers on monocytes and AMs with or without 10μM BeSO_4_ stimulation. 4a. Expression of phenotypic makers on monocytes. Upper Left. monocytes CD86(%); Upper Right. monocytes HLA DR MFI. 4b. Expression of phenotypic makers on AMs. Lower Left. AMs CD16 (%); Lower middle. AMs CD86(%); Lower Right. AMs HLA DR(%). Bars in a and b show the mean +/- SEM. *p<0.05, **p<0.01, ***p<0.001.

The phenotypic pattern of circulating monocytes from PBMC and AMs were altered after 10μM BeSO_4_ treatment for 24h (Fig. 4). The expression of CD86 on monocytes was increased significantly in CBD, BeS-NS and HS participants (p<0.05, Fig. 4A) after stimulation with 10μM BeSO_4_ for 24h, while no change in the expression of CD14, CD16 and HLA-DR was noted before and after treatment with BeSO_4_ (data not shown). Based on the MFI, increased HLA-DR was observed on circulating monocytes after treatment in all three groups (Fig. 4A). The expression of CD16 on AMs from both CBD and BeS-NS subjects was downregulated significantly after stimulation with 10μM BeSO_4_ for 24h, while the expression of CD86 and HLA-DR was unaltered with Be treatment in all subjects (Fig. 4B).

### Phagocytosis by CBD and BeS-NS AMs is significantly downregulated after stimulation with BeSO_4_

**Fig 5:**
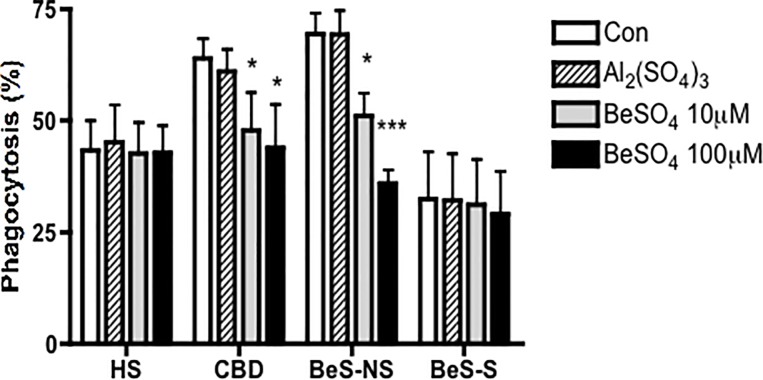
The phagocytosis of AMs in CBD and BeS-NS were downregulated significantly after stimulation with BeSO_4_ for 24h. Bars show the mean +/- SEM. *p<0.05, ***p<0.001.

Investigation of phagocytic capacity of adherent AMs from CBD and BeS-NS revealed higher phagocytosis compared with HS and BeS-S (p<0.05) (Fig. 5). However, after the 10μM and 100μM BeSO_4_ treatment for 24h, only CBD and BeS-NS AMs demonstrated a significant reduction in the percentage of AMs displaying phagocytosis, with the AMs from BeS-NS demonstrating a 26.4% reduction after treatment with 10μM BeSO_4_ (p<0.05) and a 48.3% reduction with 100μM BeSO_4_ (p<0.0001), while with the AMs from CBD demonstrating a 25.1% reduction after treatment with 10μM BeSO_4_ (p<0.05) and a 31.4% reduction with 100μM BeSO_4_ (p<0.05). No difference was found in the phagocytosis by the HS and BeS-S AMs with Be treatment.

## Discussion

In the present work we have demonstrated that the circulating CD14^dim^CD16+ monocytes, which represent a pro-inflammatory subpopulation, are present at a lower frequency in PBMCs than the lung of CBD and BeS-NS patients. We have also shown that AMs of CBD and BeS-NS display an activated cell surface phenotype with higher frequencies of the markers CD40, CD86 and HLA-DR in contrast to AMs of HS and BeS-S. Furthermore, the AMs from CBD and BeS-NS have an increased phagocytic ability compared to HS and BeS-S AMs. Interestingly, Be treatment down regulates the CD16 expression on AMs as well as the phagocytic capacity of CBD and BeS-NS AMs. Collectively, these findings may indicate that CD14^dim^CD16+ monocytes are lower in peripheral blood of CBD and BeS-NS patients as they compartmentalize and differentiate into mature and activated CD16+ AMs in the lung. Furthermore, these results may also suggest that Be exposure alters the balance between an innate and acquired immune response, with the promotion of activated AMs with reduced phagocytic capacity, which in turn result in a Th1 inflammatory response and deficient antigen clearance in the lung.

CD14+CD16+ monocytes are a subpopulation of cells that have acquired features consistent with mature tissue macrophages while in the circulation [16–17]. The CD14^bright^CD16+ monocyte subpopulation has been reported to contain the majority of IL-10-producing cells [18] and to produce high levels of proinflammatory cytokines, such as TNF-α and IL-1β. In addition, this monocyte subpopulation has been noted to be selectively expanded in a number of inflammatory processes, including coronary artery disease [19], severe asthma [31], and sarcoidosis [13]. In contrast, CD14^dim^CD16+ monocytes were recently shown to have high migratory but only limited phagocytic potential [20], and did not produce ROS or cytokines in response to cell surface toll-like receptors. Instead, they selectively produced TNF-α, IL-1β, and CCL3 in response to viruses and immune complexes containing nucleic acids, via a proinflammatory TLR7-TLR 8-MyD88-MEK pathway [20]. In the present study, we demonstrated that CD14^dim^CD16+ cells were at decreased frequency in PBMCs of both BeS-NS and CBD compared to HS, while CD14^bright^CD16+ monocytes did not differ between groups. In addition, we observed that the expression of HLA-DR was higher in both CD14^bright^CD16+ and CD14^dim^CD16+ monocyte subsets, suggesting that these cells are activated. We therefore hypothesized that blood CD14^dim^CD16+ monocytes compartmentalized to the lung and differentiated into AMs in CBD and BeS-NS, accounting for the decrease in the CD14^dim^CD16+ monocyte subsets we observed in blood cells. In support of this hypothesis, we investigated the phenotype of AMs derived from BAL. Indeed, our data demonstrate that CD14^dim^CD16+ cells were increased in CBD AMs, while CD14^bright^CD16+ cells were increased in AMs of BeS, compared to HS. Not surprising, the CBD AMs displayed an activated cell surface phenotype, with increased expression of CD16, CD40, CD86 and HLA-DR compared to HS AMs. As a robust Th1 cytokine response is noted in CBD [32, 33], our data supports that these cells contribute to the proinflammatory cytokine production in CBD lung, similar to that noted for sarcoidosis [34]. Surprisingly, the fresh AMs cells from BeS-NS demonstrated a similar patter to those of CBD, with increased expression of activation markers despite the fact that BeS-NS subjects are known to have normal BAL WBC counts and lymphocyte %, reduced or absent lung Th1 cytokine production as well as normal pathology (similar to data not shown from our subjects in this study). Since there are no difference in the AM phenotype between BeS-NS and CBD, there must be other differences in BeS-NS and CBD AMs, such as the presence of pivotal immune response genes and networks, which impact the Th1 immune response to Be and account for progression from BeS-NS to CBD.

The AMs are not thought to be the most important cell type involved in antigen presentation in the lower respiratory tract. Lung dendritic cells are thought to be the crucial APC, picking up the antigen and carrying it to local lymph nodes, where a specific immune response can be initiated [35]. AMs may act as silencers of an immune response in the lung [36]. However, in some diseases such as asthma, hypersensitivity pneumonitis (HP), and sarcoidosis [37–39], AMs become involved in the maintenance and further expansion of the immune response in the target organ, the lung. Previous studies from our group have shown that CBD BAL cells induce T cell proliferation and produce Th1-type cytokines, including TNF-α and IFN-γ in response to Be-stimulation [43]. We speculated that BeS-NS AMs would have been active in silencing the immune response to Be and perpetuated maintenance of sensitization versus progression to CBD. Our finding of overexpression of CD16, CD40, CD86 and HLA-DR on AMs in CBD suggests increased AMs activation which in turn an increased Th1 cytokine production and immune response in CBD, likely with increased antigen presentation to T cells. Since we did not find differences in the AM activation between BeS-NS and CBD, this might suggest that AMs in BeS-NS and CBD are not able to silence the Be-immune response and instead perpetuate inflammation in the lung once started. Alternatively, other mechanisms besides the AM phenotype and activation may downregulate the immune response to Be in the BeS-NS lung.

We found that the phenotypic pattern of circulating monocytes and AMs were altered after Be treatment. Specifically, following Be challenge, the %CD86 on circulating monocytes increased in all the groups and %CD16 on AMs in both BeS-NS and CBD decreased (Fig. 4A and 4B). The HLA-DR MFI on circulating monocytes also increased with Be exposure (Fig. 4A). Other costimulatory markers were not altered with Be-stimulation. These results indicate that Be has the potential to trigger innate AM immune cell activation whether from CBD, BeS-NS or non-CBD individuals. It is likely that this alteration contributes to the induction of non-specific lung inflammation and injury, as well as to the development of BeS-NS in genetically susceptible individuals. Supporting this notion, prior studies have demonstrated that Be exposure in non-diseased human and animal cell models stimulates a variety of cellular responses, including cell migration [40], and cytokine regulation [41]. However, not all cells respond to Be in the same manner, as our data indicate that only CBD and BeS-NS AMs and circulating monocytes demonstrate a reduction in CD16, and thus a different phenotypic pattern with exposure to Be.

To further better define the impact of the loss in CD16 expression with Be challenge, we evaluated alterations in the phagocytic capability of AMs after Be treatment, as cross-linking of CD16 (a member of the of FcγR family) on macrophages initiates phagocytosis. We found that the phagocytic capacity of both BeS-NS and CBD AMs was downregulated, while no difference was found in the phagocytic capacity of HS and BeS-S with Be stimulation. While the reduced phagocytic ability of BeS-NS and CBD AMs is likely related to CD16 reduction, our previous work has shown that Be-stimulates macrophage apoptosis. Specifically, Be stimulates apoptosis in mouse and human macrophage cell lines and in CBD and BeS-NS BAL macrophages [4, 42] which is dependant on the activation of intracellular caspases and the up-regulation of ROS, but independent of Be-stimulated TNF-α production [5]. CD16-positive monocyte-derived macrophages showed a higher phagocytosis for opsonized Escherichia coli [43]. We speculate that this results in CD16 reduction on macrophages as they undergo apoptosis. In addition, the loss of surface expression of CD16 will contribute to reduced phagocytosis. Meanwhile, the phagocytic ability of AMs in HS did not change, which correlates with the lack of alteration in CD16 noted in these cells. Our data may provide at least a partial explanation for the protective effect of smoking on non-infectious granulomatous inflammatory diseases, such as sarcoidosis, HP and Crohn’s disease [22–25], as smoking reversed the expression of AMs activated surface markers to normal (Fig. 3) and reduced the expression of costimulatory molecules and phagocytosis. These changes may in turn inhibit the AMs ability to mount a Be-specific immune and inflammatory response in the lung as well as impair antigen presentation and T cell activation in the lung. That is maybe, at least in part, responsible for the protection that smokers have against developing the granulomatous disease CBD.

In summary, our data suggest that there is an inappropriate Be-stimulated immune response in CBD and BeS-NS with alterations in an AM phenotype that is activated compartmentalized to the lung. These AMs demonstrate reduced phagocytic ability likely resulting in the inability to clear a Be antigen in BeS-NS and CBD. We speculate that the Be-stimulation of a more mature CD16+ AMs in turn results in increased Th1 cytokine and chemokine production, and perpetuates the Be immune response in CBD. While it is clear that Be stimulates an innate immune response with the activation of AMs markers in HS, ultimately there must be an alteration in the balance between an innate and acquired immune response to develop BeS-NS and or CBD; this imbalance may result from deficient antigen clearance with reduced phagocytosis and promotion of Th1 cytokine inflammatory responses in the lung. The latter alterations are likely dependent on AMs in contrast to circulating monocytes. However, progression from CBD and BeS-NS likely result from differences in these cells (and or others) that are beyond what we demonstrated in our study, since we found no significant difference in AMs activation and phenotype between BeS-NS and CBD. Understanding the scope of the dysregulated immune response from Be-stimulated AMs in BeS-NS and CBD and defining ways of reversing this immune response is a focus of our future studies to prevent and/or treat CBD.
